# Dual-energy CT quantitative parameters for evaluating Immunohistochemical biomarkers of invasive breast cancer

**DOI:** 10.1186/s40644-020-00370-7

**Published:** 2021-01-07

**Authors:** Xiaoxia Wang, Daihong Liu, Xiangfei Zeng, Shixi Jiang, Lan Li, Tao Yu, Jiuquan Zhang

**Affiliations:** Department of Radiology, Chongqing University Cancer Hospital, School of Medicine, Chongqing University, Chongqing, 400030 People’s Republic of China

**Keywords:** Dual-energy CT, Quantitative parameters, Invasive breast cancer, Immunohistochemical biomarkers

## Abstract

**Background:**

Estrogen receptor (ER), progesterone receptor (PR), human epidermal growth factor receptor 2 (HER2) and Ki67 are the most useful immunohistochemical biomarkers of invasive breast cancer. The purpose of this study is to investigate the possibility of quantitative parameters derived from dual-energy CT (DECT) to discriminate immunohistochemical biomarkers of invasive breast cancer.

**Methods:**

This prospective study enrolled 120 patients with invasive breast cancer who underwent preoperative contrast-enhanced DECT for staging purposes from June 2019 to January 2020. DECT quantitative parameters, including normalized iodine concentration (NIC), the slope of the spectral Hounsfield unit curve (λ_Hu_), and the normalized effective atomic number (nZ_eff_), were obtained from reconstructed images. DECT quantitative parameters were compared with the expression status, and the correlations with the value of immunohistochemical biomarkers were evaluated. Inter-observer reproducibility analysis was performed to assess the measurement reproducibility of quantitative parameters. The diagnostic performance of the quantitative parameters was analyzed by receiver operating characteristic curve.

**Results:**

The ER-negative group tended to display higher venous phase NIC and nZ_eff_ compared with the ER-positive group (individually, *p* = 0.003, 0.011; area under the curve [AUC] of 0.65, 0.60). The PR-negative group demonstrated higher arterial and venous phase NIC compared with the PR-positive group (individually, *p* = 0.022, 0.005; AUC of 0.63, 0.65). NIC was correlated negatively with the value of ER and PR expression (*r* = − 0.175 ~ − 0.265, *p* = 0.002 ~ 0.042). The HER2-positive group tended to display higher venous phase nZ_eff_ than the HER2-negative group (*p* = 0.022; AUC of 0.59). The Ki67 high-proliferation group demonstrated higher arterial phase, venous phase NIC and nZ_eff_ than the Ki67 low-proliferation group (*p* < 0.001 ~ 0.005; AUC of 0.67 ~ 0.75). Both the NIC and nZ_eff_ were correlated positively with the value of Ki67 (*r* = 0.240 ~ 0.490, *p* < 0.001 ~ 0.014).

**Conclusions:**

NIC and nZ_eff_ derived from DECT could be used to discriminate expression status and may associate with the value of immunohistochemical biomarkers of invasive breast cancer.

## Introduction

Breast cancer is a heterogeneous tumor that represents the accumulation of complex genetic alterations and can be divided into different subtypes according to the receptor status. The most useful receptors in breast cancer cells, which determine therapy strategy, are estrogen receptor (ER), progesterone receptor (PR) and human epidermal growth factor receptor 2 (HER2). ER-positive and PR-positive cases have a lower risk of mortality than ER-negative and/or PR-negative cases, while HER2-positive breast cancers tend to be more aggressive and have a poorer prognosis than HER2-negative breast cancer [1, 2]. Several studies [3, 4] have proved the prognostic value of the Ki67 index in patients with breast cancer. Preoperative phenotyping of breast cancer is critical because it may predict the response to neoadjuvant chemotherapy and provide optimized strategies for patient-tailored therapy.

Currently, multiple quantitative parameters derived from dual-energy CT (DECT), including normalized iodine concentration (NIC), the slope of the spectral Hounsfield unit (HU) curve (λ_Hu_, in Hounsfield unit per kiloelectron-volt), and the normalized effective atomic number (nZ_eff_), have attracted wide research interest in tumors, such as head [5, 6], lung [7–9], liver [10, 11], kidney [12, 13], and other organ imaging studies [14–16]. DECT has been proven to be an effective method to evaluate neoangiogenesis and has been increasingly applied in tumor detection and characterization [17]. Breast cancer development typically results in angiogenesis, which increases the formation of small vessels in and near the tumor [18, 19].

Clinicians usually use contrast-enhanced CT scans of the thorax for the evaluation of potential lung metastasis or underlying lung tuberculosis [20]. However, routine thorax CT is unable to characterize the primary breast tumor precisely due to its relatively low resolution of soft tissue, limited imaging mode and qualitative parameters [21]. A few studies using iodine-based material decomposition images have shown that the iodine concentration was significantly higher in tumors than in normal breast tissue and pectoral muscle [22]. A recent study [23] demonstrated the feasibility of DECT for locoregional staging of breast cancer, and the iodine concentration of invasive non-special carcinoma was higher than that of ductal carcinoma in situ and benign tumors. Therefore, we attempted to correlate the quantitative parameters derived from DECT with immunohistochemical factors of breast cancer.

The purpose of this study was to investigate the possibility of quantitative parameters derived from DECT to discriminate immunohistochemical biomarkers of invasive breast cancer.

## Materials and methods

### Patient characteristics

This prospective study was approved by the institutional review board, and written informed consent was collected from every participant. From June 2019 to February 2020, 120 consecutive women were enrolled, with a median age of 53.3 ± 9.9 years (range, 32 ~ 87 years). Ninety-seven women who were pathological diagnosed breast cancer, were instructed to undergo DECT for evaluation of potential lung metastasis, while twenty-three women who were suspected of having breast cancer by on mammography or/and ultrasonography or/and MRI, were instructed to undergo DECT for evaluation of lung tuberculosis or other inflammation. Finally, the lesions were confirmed by histopathology as invasive breast cancer after the examination. Inclusion criteria included a first diagnosis of breast cancer, breast masses with a shortest diameter larger than 1 cm and visibility on DECT-enhanced images, women who had not undergone biopsy of the breast mass within 1 week before the initial CT examination, and women who had no history of chemotherapy or radiation therapy in the breast space. The exclusion criteria for DECT were based on the clinical guidelines of contrast-enhanced CT at our institution: impaired kidney function (glomerular filtration rate < 30 ml/min), severe contrast media allergy, and inability to give informed consent for the CT examination. Patient demographics and histopathological diagnosis data for each patient were extracted from the hospital’s electronic medical records.

We reviewed the immunohistochemistry diagnosis data from the hospital’s electronic medical records. The immunohistochemical results of ER, PR and HER2 were classified as positive or negative [24]. ER or PR positivity was defined as ≥1% nuclear immunostaining. HER2 overexpression was considered in 3+ immunohistochemical staining or 2+ immunohistochemical staining and HER2 gene amplification in silver-stained in situ hybridization. The Ki-67 index was assessed as the percentage of immunoreactive tumor cells, and a cut-off value of 20% was used to define the low- and high-proliferation tumor groups [25].

### DECT image acquisition

Image data were acquired on a 2.5 generation dual-source CT unit (SOMATOM Drive, Siemens Healthineers, Forchheim, Germany) in dual-energy mode through two X-ray tubes with different kV tube voltages (tube A, 100 kV; tube B, Sn 140 kV) using a tin filter for the high-voltage tube. Automatic exposure control (CARE Dose 4D, Siemens Healthineers) was used in all scans. The settings for the scanners were as follows: collimation, 64 × 0.6 mm; rotation time, 0.28 s; pitch, 0.55; reference tube current time product, 71 mAs for the 100-kVp tube and 60 mAs for the Sn140-kVp tube; reformatted section thickness, 1.5 mm; reformatted section increment, 1.5 mm.

All patients were scanned craniocaudally in the supine position with the bilateral arms elevated in close contact with the head. The whole chest was scanned from the superior aperture of the thorax to the inferior edge of the costophrenic angle during a deep-inspiratory breath hold, which covered the breast and axillary area. For contrast-enhanced scanning, an iodinated nonionic contrast media (Ioversol, 320 mg/ml iodine, HENGRUI Medicine, Jiangsu, China) was administered through the right or left ulnar vein by a dual-head injector. The dosage was 1.5 ml/kg with a flow rate of 2.5 ml/sec, followed by a bolus injection of 30 ml of saline given at the same flow rate. The side of the ulnar vein that was injected was selected to be contralateral to the suspected breast lesion to avoid a beam hardening artifact of the axillary vein. After the injection, the arterial phase scans were started using a bolus-tracking technique with a threshold of 100 HU in the descending aorta and an additional delay of 10 s. The scan delay time for the venous phase scanning was 25 s after the end of the arterial phase scanning.

### DECT image analysis

DECT data were analyzed by using viewer software on a syngo.via workstation (syngo.via VB20A, Dual Energy, Siemens Healthineers, Forchheim, Germany). Standard linear-blended images were reconstructed by applying a blending factor of 0.5 (M_0.5; 50% of the low kV and 50% of the high-kV spectrum). DECT quantitative parameters were measured by two radiologists (X.X.W, with 6 years of experience in breast and chest diagnostic imaging, and X.F.Z, with 2 years of experience in postreconstruction imaging) who were blinded to the immunohistochemical results of invasive breast cancer, by placing a circular region of interest, 1 cm^2^ in size, excluding any area of obviously gross necrosis, calcification or large vessels. Quantitative parameters, including the iodine concentration (in milligrams per cubed centimeter) and the effective atomic number, were divided by the iodine concentration and effective atomic number of aorta, respectively, to obtain the normalized iodine concentration (NIC) and normalized effective atomic number (nZ_eff_). The slope of the spectral Hounsfield unit curve (λ_Hu_ in Hounsfield unit per kiloelectron-volt) was calculated as follows [26]:

$$ \mathsf{\lambda Hu}=\left(\mathsf{HU}\mathsf{4}\mathsf{0keV}-\mathsf{HU}\mathsf{7}\mathsf{0keV}\right)/\mathsf{30}\;\mathsf{keV} $$

### Statistical analysis

Statistical analyses were performed using commercially available statistical software (IBM SPSS Statistics, version 22.0). Inter-observer agreement was calculated with quadratic weighted Cohen’s kappa coefficients, with values of ≥0.81 indicating excellent, 0.61 ~ 0.80 substantial, 0.41 ~ 0.60 moderate, 0.21 ~ 0.40 fair, and ≤ 0.20 poor agreement. Quantitative variables are expressed as the mean ± standard deviation. In the univariable analyses, continuous data were compared using the independent sample *t* test. Receiver operating characteristic (ROC) curve analysis was used to evaluate the diagnostic capacity of DECT quantitative parameters. The sensitivity, specificity, and accuracy with 95% confidence intervals were calculated, and the optimal threshold was determined by the Youden index. The *Spearman* correlation test was used to correlate DECT quantitative parameters with the value of immunohistochemical biomarkers in invasive breast cancer. The degree of correlation was classified as a direct correlation or an inverse correlation depending on whether *r* was a positive value or a negative value, respectively. The level of significance was defined as *p* < 0.05.

## Results

### Patient characteristics

Ultimately, 120 participants (53.3 ± 9.9 years; range, 32 ~ 87 years) who had invasive breast cancer with DECT images were included in our study. Another 120 participants were excluded because they had no/incomplete pathological information (*n* = 23), received a mass biopsy within 1 week before CT scanning (*n* = 33), had benign tumors (*n* = 22), had a breast mass invisible/shortest diameter < 1 cm (*n* = 12), had a breast mass exceeding the field of view due to obesity (*n* = 7), and had received neoadjuvant chemotherapy before CT scanning (n = 23). Demographic characteristics and the immunohistochemical biomarkers distribution in our study population are provided in Table 1.

**Table 1:** Summary of Demographic Characteristics

Characteristic	Number
No. of Participants	120
Age, Mean ± SD, years (Range)	53.3 ± 9.9 years (range, 32 ~ 87 years)
Menstruation State	
Premenopausal women	34
Postmenopausal women	69
Perimenopausal women	17
Tumor size, Mean ± SD, cm	2.96 ± 1.38 cm
Tumor size	
≤ 2 cm	32
> 2 cm	88
ER	
positive	86
negative	34
PR	
positive	53
negative	67
HER2	
positive	47
negative	73
Ki67	
low-proliferation	70
high-proliferation	50

Note. *SD* standard deviation, *ER* estrogen receptor, *PR* progesterone receptor, *HER2* human epidermal growth factor receptor 2

**Fig. 1:**
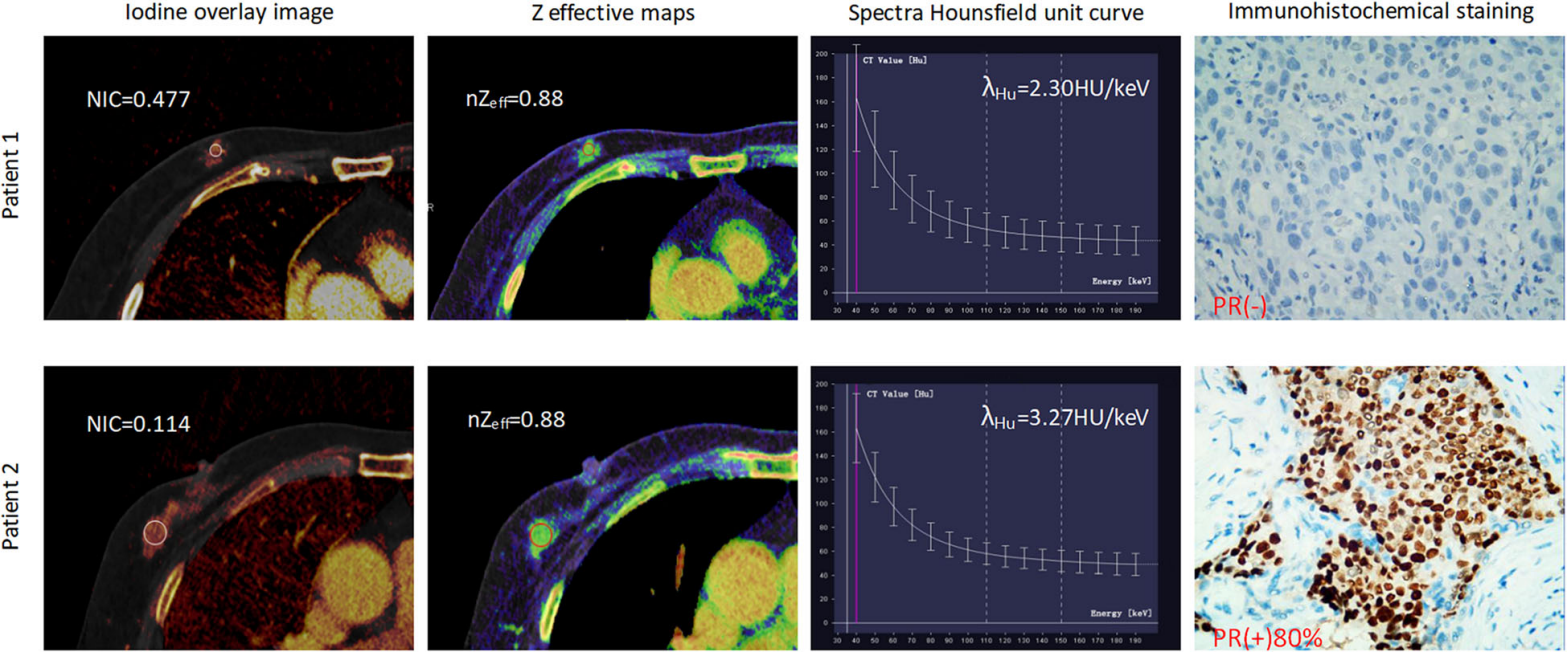
The dual-energy CT quantitative parameters and immunohistochemical staining in two women with invasive breast cancer. Patient 1 was a 50-year-old woman who was PR- negative, while patient 2 was a 47-year-old woman who was PR-positive. NIC = normalized iodine concentration; nZ_eff_ = normalized effective atomic number; λ_Hu_ = slope of the spectral Hounsfield unit curve; PR = progesterone receptor

**Fig. 2:**
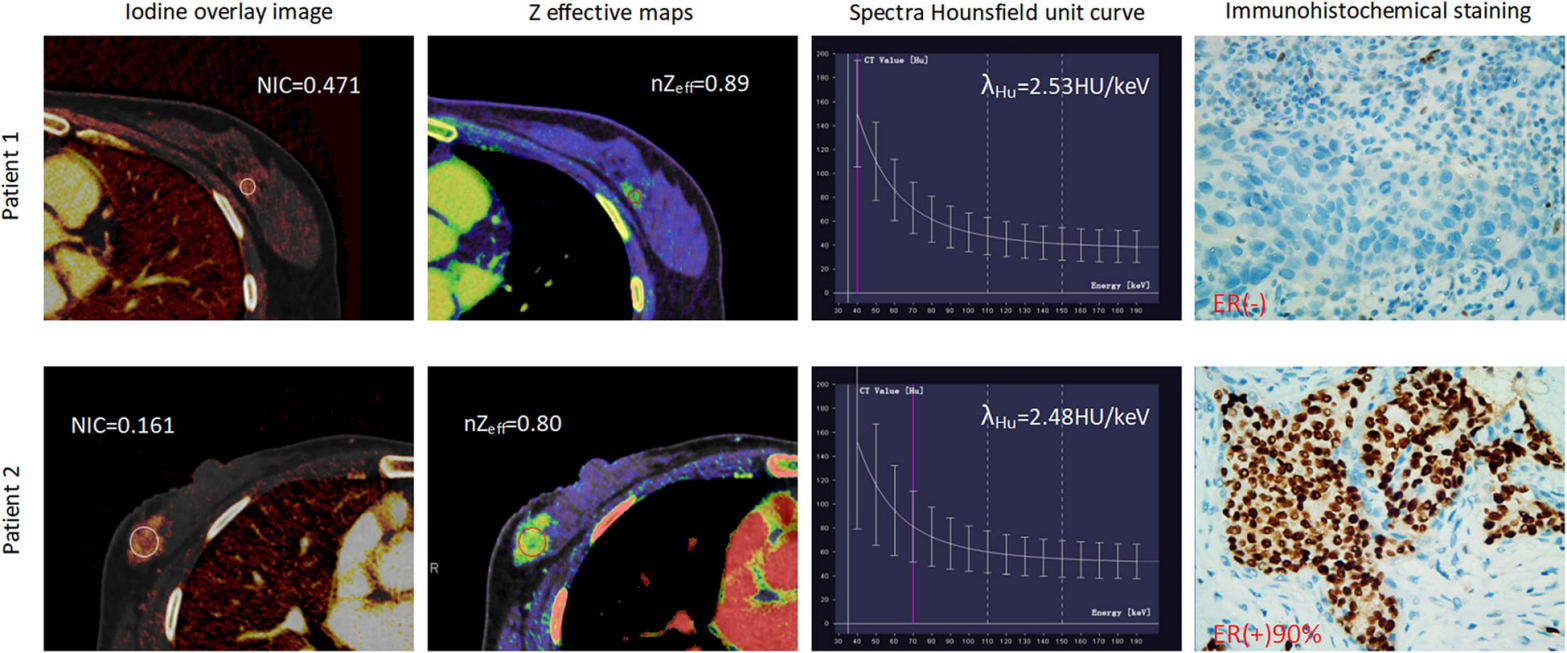
The dual-energy CT quantitative parameters and immunohistochemical staining in two women with invasive breast cancer. Patient 1 was a 45-year-old woman who was ER- negative, while patient 2 was a 52-year-old woman who was ER-positive. NIC = normalized iodine concentration; nZ_eff_ = normalized effective atomic number; λ_Hu_ = slope of the spectral Hounsfield unit curve; ER = estrogen receptor

**Fig. 3:**
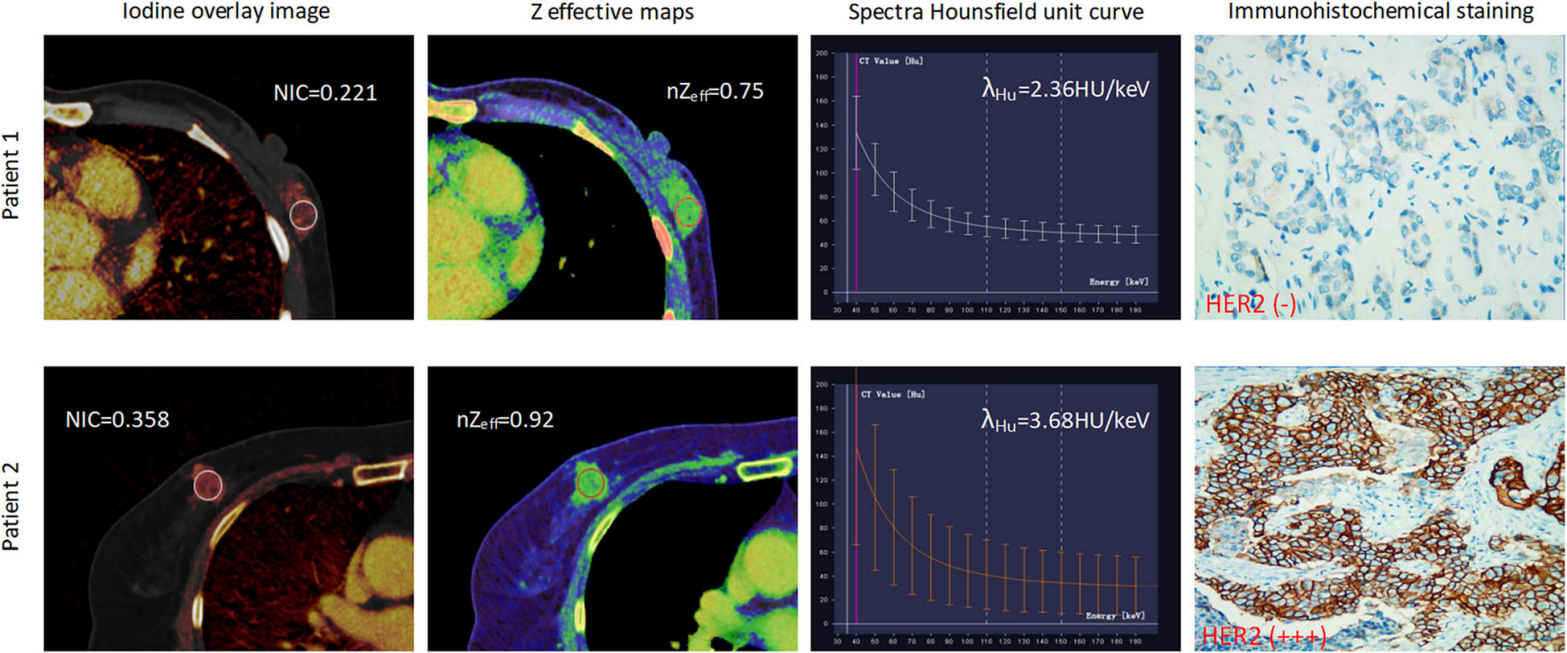
The dual-energy CT quantitative parameters and immunohistochemical staining in two women with invasive breast cancer. Patient 1 was a 56-year-old woman who was HER2-negative, while patient 2 was a 50-year-old woman who was HER2-positive. NIC = normalized iodine concentration; nZ_eff_ = normalized effective atomic number; λ_Hu_ = slope of the spectral Hounsfield unit curve; HER2 = human epidermal growth factor receptor 2

**Fig. 4:**
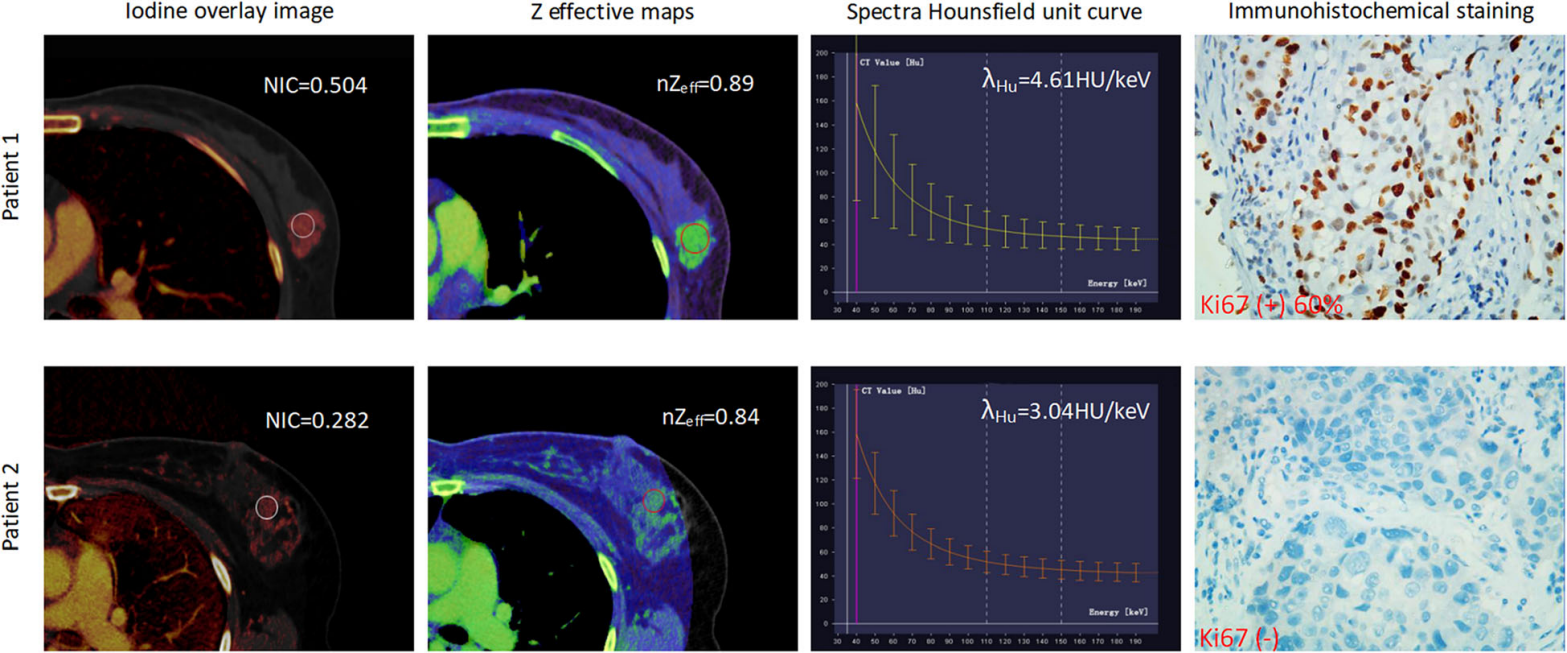
The dual-energy CT quantitative parameters and immunohistochemical staining in two women with invasive breast cancer. Patient 1 was a 51-year-old woman who was Ki67 high-proliferation, while patient 2 was a 46-year-old woman who was Ki67 low-proliferation. NIC = normalized iodine concentration; nZ_eff_ = normalized effective atomic number; λ_Hu_ = slope of the spectral Hounsfield unit curve

The mean diameter of total tumor sizes was 2.96 ± 1.38 cm, and tumors were larger than 2 cm in 88 cases and 2 cm or smaller in 32 cases. The 120 cases of invasive breast cancer were confidently classified as ER-positive (*n* = 86) vs ER-negative (*n* = 34), PR-positive (*n* = 53) vs PR-negative (*n* = 67), HER2-positive (*n* = 47) vs HER2-negative (*n* = 73), and Ki67 low-proliferation (*n* = 70) vs Ki67 high-proliferation (*n* = 50). Representative iodine overlay images, Z effective maps, graphs of spectra Hounsfield unit curves and immunohistochemical staining for patient with PR-negative and PR-positive (Fig. 1), ER-negative and ER-positive (Fig. 2) HER2-negative and HER-positive (Fig. 3), Ki67 high-proliferation and Ki67 low-proliferation (Fig. 4) are illustrated.

**Fig. 5:**
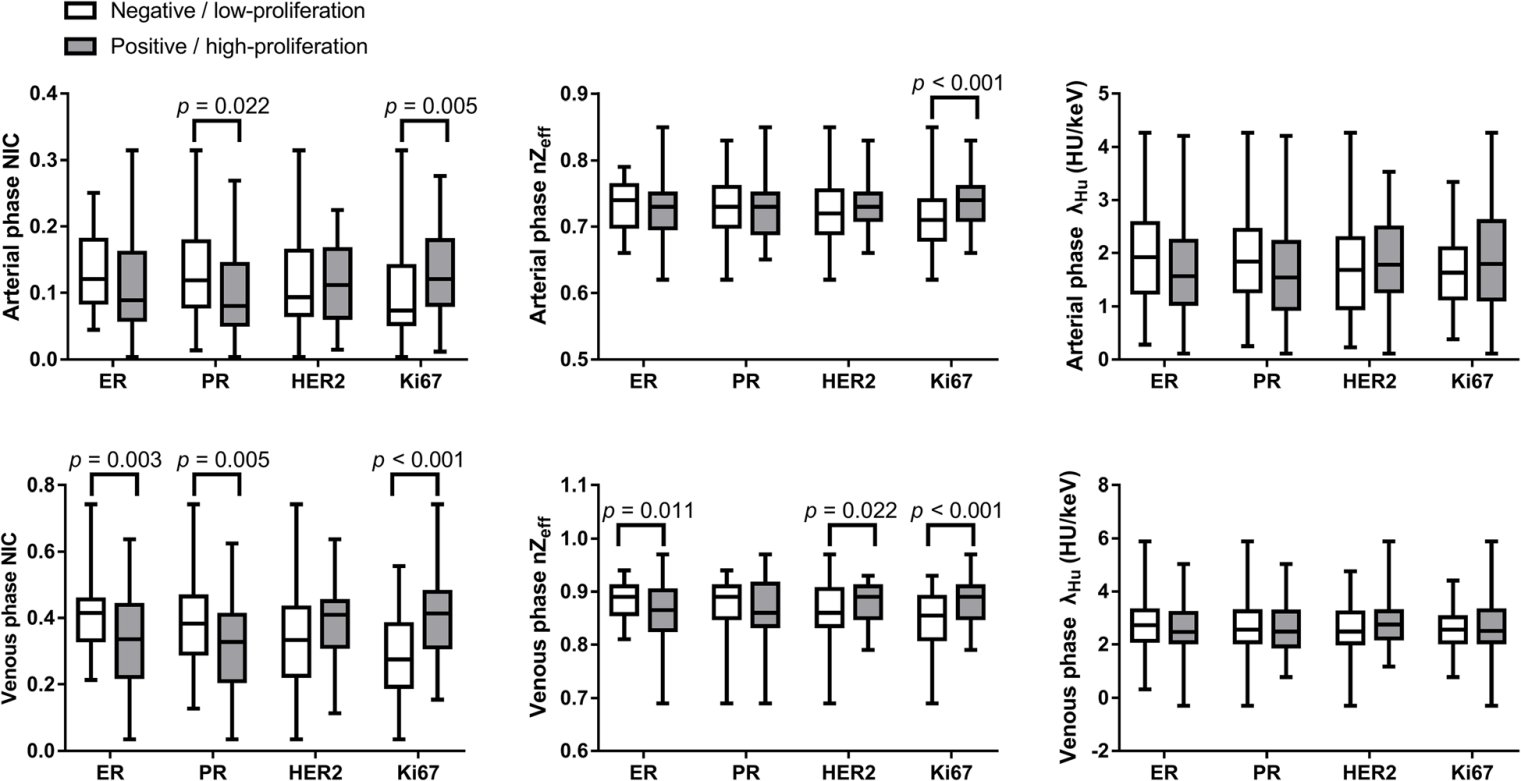
Subdivision of groups by the expression status of immunohistochemical biomarkers. **a–c** Box plots show normalized iodine concentration (**a**), normalized effective atomic number (**b**), and the slope of the spectral Hounsfield unit curve (**c**), as observed on DECT images. Horizontal lines within boxes denote median values, and vertical lines denote minimum and maximum values. ER = estrogen receptor, PR = progesterone receptor, HER2 = human epidermal growth factor receptor 2

The mean CT dose index volume was 15.43 ± 4.89 mGy, and the mean dose length product was 470.22 ± 129.83 mGy cm for each woman. Summary statistics of DECT quantitative parameters among immunohistochemical biomarkers of invasive breast cancer are presented in Table 2 and Fig. 5. Inter-observer agreement for the evaluation of DECT quantitative parameters ranged between substantial and excellent, with kappa values of 0.740 ~ 0.938. The area under the ROC curve (AUC), sensitivity, specificity and accuracy with 95% confidence intervals for the quantitative parameter thresholds are summarized in Table 3. Correlations of DECT quantitative parameters with the values of immunohistochemical biomarkers in invasive breast cancer are summarized in Table 4.

**Table 2:** Correlation of Dual-energy CT Quantitative Parameters with Immunohistochemical Biomarkers

Parameters	ER		*p* value	PR		*p* value	HER2		*p* value	Ki67		*p* value
	Positive	Negative		Positive	Negative		Positive	Negative		High-proliferation	Low-proliferation	
Arterial phase NIC	0.109 ± 0.067	0.133 ± 0.055	0.069	0.101 ± 0.063	0.128 ± 0.064	0.022	0.114 ± 0.058	0.117 ± 0.069	0.850	0.130 ± 0.063	0.096 ± 0.064	0.005
Venous phase NIC	0.336 ± 0.143	0.411 ± 0.133	0.003	0.317 ± 0.138	0.388 ± 0.133	0.005	0.381 ± 0.120	0.342 ± 0.149	0.138	0.409 ± 0.129	0.283 ± 0.120	<0.001
Arterial phase nZ_eff_	0.72 ± 0.04	0.73 ± 0.04	0.164	0.72 ± 0.03	0.73 ± 0.04	0.437	0.73 ± 0.03	0.72 ± 0.05	0.077	0.74 ± 0.03	0.71 ± 0.05	<0.001
Venous phase nZ_eff_	0.86 ± 0.06	0.88 ± 0.03	0.011	0.86 ± 0.06	0.87 ± 0.04	0.425	0.88 ± 0.03	0.86 ± 0.06	0.022	0.88 ± 0.04	0.85 ± 0.06	<0.001
Arterial phase λ_Hu_ (HU/keV)	1.71 ± 0.84	1.94 ± 0.88	0.181	1.64 ± 0.85	1.88 ± 0.84	0.127	1.86 ± 0.76	1.72 ± 0.90	0.358	1.87 ± 0.96	1.64 ± 0.67	0.129
Venous phase λ_Hu_ (HU/keV)	2.63 ± 0.98	2.75 ± 1.01	0.567	2.64 ± 0.85	2.68 ± 0.98	0.848	2.79 ± 0.95	2.58 ± 1.01	0.252	2.72 ± 1.10	2.58 ± 0.81	0.421

Note—*NIC* normalized iodine concentration; *nZ*_*eff*_ normalized effective atomic number; *λ*_*Hu*_ slope of the spectral Hounsfield unit curve; *HU* hounsfield unit; *ER* estrogen receptor; *PR* progesterone receptor; *HER2* human epidermal growth factor receptor 2

**Table 3:** Receiver Operating Characteristic Curve Analysis of DECT Quantitative Parameters for the DifferentialDiagnosis of Immunohistochemical Factors in Invasive Breast Cancer

Parameter	AUC	Threshold of Parameter	Sensitivity (%)	Specificity (%)	Accuracy (%)
ER positive vs. negative					
Venous phase NIC	0.65 [0.56, 0.75]	0.293	88.4 (76/86) [79.2, 94.0]	41.2 (14/34) [25.1, 59.2]	75.0 (90/120) [66.5, 81.9]
Venous phase nZ_**eff**_	0.60 [0.49, 0.70]	0.87	70.9 (61/86) [60.0, 80.0]	50.0 (17/34) [32.8, 67.2]	65.0 (78/120) [56.1, 73.0]
PR positive vs. negative					
Arterial phase NIC	0.63 [0.53, 0.73]	0.082	75.5 (40/53) [61.4, 85.9]	50.7 (34/67) [38.4, 61.0]	61.7 (74/120) [52.7, 69.9]
Venous phase NIC	0.65 [0.55, 0.75]	0.356	62.3 (33/53) [47.9, 74.9]	62.7 (42/67) [50.0, 73.1]	62.5 (75/161) [53.6, 70.7]
HER2 positive vs. negative					
Venous phase nZ_**eff**_	0.59 [0.49, 0.69]	0.87	70.2 (33/47) [54.9, 82.2]	53.4 (39/73) [41.4, 65.0]	60.0 (72/120) [51.1, 68.3]
Ki67 low- vs. high-proliferation					
Arterial phase NIC	0.67 [0.57, 0.77]	0.079	80.0 (56/70) [68.4, 88.3]	54.0 (27/50) [39.5, 67.9]	69.2 (83/120) [60.4, 76.8]
Venous phase NIC	0.75 [0.67, 0.84]	0.281	88.6 (62/70) [78.2, 94.6]	52.0 (26/50) [37.6, 66.1]	73.3 (88/120) [64.8, 80.5]
Arterial phase nZ_eff_	0.70 [0.60, 0.79]	0.73	65.7 (46/70) [53.3, 76.4]	62.0 (31/50) [47.2, 75.0]	64.2 (77/120) [55.3, 72.2]
Venous phase nZ_eff_	0.67 [0.57, 0.77]	0.86	74.3 (52/70) [62.2, 83.7]	50.0 (25/50) [35.7, 64.3]	64.2 (77/120) [55.3, 72.2]

Note—Data in parentheses are numerator/denominator; data in brackets are 95% confidential intervals. *NIC* normalized iodine concentration; *λ*_*Hu*_ slope of the spectral Hounsfield unit curve; *nZ*_*eff*_ normalized effective atomic number; *HU* Hounsfield unit; *AUC* area under the curve

**Table 4:** Correlations of DECT Quantitative Parameters with the Value of Immunohistochemical Biomarkers in Invasive Breast Cancer

Parameters	*r* value	*p* value
Venous phase NIC and ER value	−0.265	0.002
Venous phase nZ_eff_ and ER value	−0.141	0.103
Arterial phase NIC and PR value	−0.175	0.042
Venous phase NIC and PR value	−0.239	0.005
Venous phase nZ_eff_ and HER2 value	0.112	0.194
Arterial phase NIC and Ki67 value	0.315	< 0.001
Venous phase NIC and Ki67 value	0.490	< 0.001
Arterial phase nZ_eff_ and Ki67 value	0.225	0.014
Venous phase nZ_eff_ and Ki67 value	0.240	0.009

Note. *NIC* normalized iodine concentration; *nZ*_*eff*_ normalized effective atomic number; *ER* estrogen receptor; *PR* progesterone receptor; *HER2* human epidermal growth factor receptor 2

### Associations between DECT quantitative parameters and hormone receptors

ER-negative tumors showed significantly higher venous phase NIC and venous phase nZ_eff_ (*p* = 0.003 and 0.011, respectively) than ER-positive tumors, with areas under the curve (AUCs) of 0.65 and 0.60, sensitivities of 88.4 and 70.9%, specificities of 41.2 and 50%, and accuracies of 75 and 65%. The value of ER expression was correlated negatively with the venous phase NIC in invasive breast cancer (*r* = − 0.265, *p* = 0.002). Likewise, PR-negative tumors showed significantly higher arterial and venous phase NIC than PR-positive tumors (*p* = 0.022 and 0.005, respectively), with AUCs of 0.63 and 0.65, sensitivities of 75.5 and 62.3%, specificities of 50.7 and 62.7%, and accuracies of 61.7 and 62.5%. The value of PR expression was correlated negatively with the arterial and venous phase NIC in invasive breast cancer (*r* = − 0.175, − 0.239, *p* = 0.042, 0.005, respectively).

### Associations between DECT quantitative parameters and HER2 receptor

HER2-positive tumors showed significantly higher venous phase nZ_eff_ (*p* = 0.022) than HER2-negative tumors, with an AUC of 0.59, a sensitivity of 70.2%, a specificity of 53.4%, and an accuracy of 60%. However, no obvious correlation (*r* = 0.112, *p* = 0.194) was observed, and no significant differences in arterial phase NIC, nZ_eff_ and λ_Hu_, venous phase NIC and λ_Hu_ were observed between HER2-positive and HER2-negative tumors (*p* = 0.077 ~ 0.850).

### Associations between DECT quantitative parameters and Ki67 value

Regarding Ki-67 expression, the Ki-67 high-proliferation group demonstrated significantly higher arterial and venous phase NIC, nZ_eff_ compared with the Ki67 low-proliferation group (*p* < 0.001 ~ 0.005), with AUCs of 0.67 ~ 0.75, sensitivities of 65.7 ~ 88.6%, specificities of 50.0% ~ 62.0%, and accuracies of 64.2% ~ 73.3%. The value of Ki67 expression was correlated positively with the NIC and nZ_eff_ values in invasive breast cancer (*r* = 0.240 ~ 0.490, *p* = 0.014 ~ < 0.001).

## Discussion

Our results showed that quantitative parameters derived from DECT could be used to discriminate immunohistochemical biomarkers of invasive breast cancer. NIC and nZ_eff_ values were associated with ER and Ki67 expression statuses, while NIC was associated with PR expression status, and the nZ_eff_ value was an indicator of HER2 in invasive breast cancer. The NIC was negatively correlated with the values of ER and PR expression, while the NIC and nZ_eff_ were positively correlated with the value of Ki-67 expression.

The hormone receptors ER and PR are known to be important prognostic factors and predictive biomarkers of endocrine therapy response to breast cancer. In this study, the ER-negative group tended to display higher venous phase NIC and venous phase nZ_eff_ compared with the ER-positive group, while the PR-negative group demonstrated significantly higher arterial and venous phase NIC compared with the PR-positive group. The NIC was correlated negatively with the values of ER and PR expression. A study [27] on the application of low-dose perfusion CT in breast cancers showed that the perfusion was significantly higher while the time to peak was significantly shorter in ER- and PR-negative breast cancers than in the positive group. This may be related to the fact that the velocity and amount of tumor vessels reflect the behavior and prognosis of the tumor. Previous research [28] has demonstrated that ER expression is associated with inhibition of angiogenesis, which would reduce perfusion. PR is generally regarded as a marker of a functional ER pathway because the transcription of the PR gene is enhanced and maintained by estrogen [29]. The distribution of iodine in the tissue is strongly related to local blood volume and vascular density [17]. Therefore, NIC may reflect the expression of ER and PR through breast cancer angiogenesis.

Breast cancer with HER2 overexpression is characterized by increased cell proliferation and angiogenesis, which is associated with metastasis progression and poor prognosis [30]. In our study, the HER2-positive group tended to display higher venous phase nZ_eff_ compared with the HER2-negative group. Material decomposition images can be used to estimate the concentration of a given material (i.e., water, gadolinium or iodine) with a given atomic mass in the region of interest, thus potentially distinguishing different tissues [31]. nZ_eff_ is a quantitative indicator of the compound atom of a compound or a mixture of various materials. A previous study [32] demonstrated that DECT allowed the confident evaluation of silicone within breast soft tissues in reconstructed images. However, the clinical significance of nZ_eff_ applied to tumors is not clear. The HER2-positive group had higher venous phase NIC than the HER2-negative group, however, these differences were not significant.

Ki67 is a biomarker considered to represent the state of tumor proliferation; a high Ki67 index is associated with adverse clinical outcomes in breast cancer patients [33]. In our study, the Ki67-positive group demonstrated significantly higher arterial phase, venous phase NIC and nZ_eff_ compared with the Ki67-negative group. Both the NIC and nZ_eff_ were correlated positively with the value of Ki67 expression. Some previous studies [34, 35] have found that the NIC values were positively correlated with Ki67 expression in rectal and gastric cancer. One possible explanation for these findings may be that high-proliferating tumors are more heterogeneous, such as breast cancer, and are characterized by complex changes, including mitosis and angiogenesis, resulting in an increase in the NIC value.

This study has several limitations. First, the number of cases was limited, and further studies with a relatively larger samples should be performed to validate the results of the present study through thresholding. Second, we focused only on invasive breast cancer. Further studies involving larger samples and wider spectra of breast lesions are warranted. Finally, direct comparisons between DECT quantitative parameters and histopathologic factors, such as cell density and microvessel density, were lacking. Therefore, the explanation for the relationship between DECT quantitative parameters and immunohistochemical biomarkers of invasive breast cancer was merely based on current findings from literature.

## Conclusions

In conclusion, NIC and nZ_eff_ derived from DECT could be used to discriminate expression status and may associate with the values of immunohistochemical biomarkers of invasive breast cancer. In particular, the NIC value was associated with ER, PR, and Ki67 status, while the nZ_eff_ value was associated with ER, HER2 and Ki67 status.

## Data Availability

The datasets used and/or analyzed during the current study are available from the corresponding author on reasonable request.

## Abbreviations

AUC: Area under the curve
ER: Estrogen receptor
HER2: Human epidermal growth factor receptor 2
HU: Hounsfield unit
keV: Kiloelectron volts
NIC: Normalized iodine concentration
nZ_eff_: Normalized effective atomic number
PR: Progesterone receptor
S: Standard deviation
Z_eff_: Z effective
λ_Hu_: Slope of the spectral Hounsfield unit curve
